# Combining the National Early Warning Score 2 with Frailty Assessment to Identify Patients at Risk of In-Hospital Cardiac Arrest: A Descriptive Exploratory Study

**DOI:** 10.3390/medicina62020311

**Published:** 2026-02-02

**Authors:** Cesare Biuzzi, Elena Modica, Alessandra Vozza, Roberto Gargiuli, Benedetta Galgani, Giovanni Coratti, Daniele Marianello, Fabio Silvio Taccone, Federico Franchi, Sabino Scolletta

**Affiliations:** 1 Urgency-Emergency Anesthesia and Intensive Care Unit, Department of Medical Science, Surgery and Neurosciences, University Hospital of Siena, 53100 Siena, Italy; cesare.biuzzi@ao-siena.toscana.it (C.B.); elena.modica@dbm.unisi.it (E.M.); alessandra.vozza@student.unisi.it (A.V.); r.gargiuli@student.unisi.it (R.G.); benedetta.galgani@ao-siena.toscana.it (B.G.); giovanni.coratti@dbm.unisi.it (G.C.); 2 Cardiothoracic and Vascular Anesthesia and Intensive Care Unit, Department of Medical Science, Surgery and Neurosciences, University Hospital of Siena, 53100 Siena, Italy; d.marianello83@gmail.com (D.M.); federico.franchi@unisi.it (F.F.); 3 Department of Intensive Care, Hôpital Universitaire de Bruxelles (HUB), Université Libre de Bruxelles (ULB), 1070 Brussels, Belgium; fabio.taccone@ulb.be

**Keywords:** in-hospital cardiac arrest (IHCA), NEWS2, Clinical Frailty Scale (CFS), Charlson Comorbidity Index (CCI), Barthel Index (BI), Early Warning Systems (EWS), Rapid Response System (RRS)

## Abstract

*Background and objectives:* In older and frail patients, in-hospital cardiac arrest (IHCA) is associated with high mortality. Early warning scores such as the National Early Warning Score 2 (NEWS2) are widely used to detect clinical deterioration, but their predictive accuracy in frail populations remains uncertain. This study aimed to assess whether integrating frailty measures with NEWS2 could better describe elderly IHCA patients. *Materials and Methods*: We conducted a single-center, retrospective observational study in adult and frail patients (≥18 years) admitted to medical and surgical wards of the University Hospital of Siena who experienced IHCA between January 2022 and January 2024. Data on demographics, such as last NEWS2 before IHCA, Clinical Frailty Scale (CFS), Barthel Index (BI), and Charlson Comorbidity Index (CCI) were retrospectively collected and analyzed. Patients were stratified into three categories, according to NEWS2: Stable (A), Potentially Unstable or Unstable (B), and Critical (C). *Results*: Seventy patients were analyzed (mean age 76.9 ± 11.0 years; 56% male). The mean pre-IHCA NEWS2 score was 6.0 ± 3.5, with 41% of patients classified as NEWS2-C, 48% classified as NEWS2-B, and 11% classified as NEWS2-A. The NEWS2-A category showed higher BI and lower CFS than NEWS2-B and NEWS2-C (*p* < 0.01), while CCI and age did not significantly differ. *Conclusions*: The association of NEWS2 with frailty scores could identify some elderly patients with limited pre-arrest physiological derangements but high frailty who suffered from IHCA. These findings provide descriptive insights that may inform monitoring strategies for “at-risk” elderly patients to help prevent IHCA.

## 1. Introduction

The global population is aging rapidly, with the median age steadily increasing and the proportion of individuals over 65 years growing. This demographic shift has profound implications for healthcare systems, as circulatory diseases remain the leading cause of death worldwide, accounting for a substantial proportion of overall mortality [1,2,3,4]. In this context, the management of acute events, such as in-hospital cardiac arrest (IHCA) in an increasingly frail and elderly population, has become a primary clinical challenge. IHCA represents a major clinical challenge, associated with high morbidity and mortality. Its incidence ranges from 1 to 3 events per 1000 hospital admissions [5], while survival rates at hospital discharge typically range between 15% and 34% [2], declining further at 30 days post event, highlighting the complexity of managing these critically ill patients [6,7]. Given that cardiac arrests are often preceded by rapid clinical deterioration, prompt and accurate identification of patients at risk has become crucial for improving outcomes and survival. Early warning systems have been developed extensively in recent decades, with particular emphasis placed on standardized early warning scores aimed at identifying patient deterioration [8,9]. Among them, the National Early Warning Score 2 (NEWS2), recommended by the Royal College of Physicians, is one of the most widely adopted [10,11]. Despite extensive validation in numerous clinical settings, the predictive accuracy and clinical effectiveness of this score in preventing IHCA remain debated, especially regarding its sensitivity and specificity, particularly in vulnerable subgroups of hospitalized patients, such as elderly individuals with multiple comorbidities and clinical frailty [12,13,14,15].

Frailty itself, a condition characterized by reduced physiological reserve, impaired functional capacity and increased vulnerability to adverse events, further complicates clinical management. Reduced compensatory mechanisms contribute to a higher risk of poor responses to acute medical events, including cardiac arrest [16,17]. The Clinical Frailty Scale (CFS) and Barthel Index (BI) provide available tools for assessing frailty [18,19]. A higher CFS and a lower BI indicate greater vulnerability to adverse outcomes, including poor recovery following IHCA [20,21]. This approach provides a quantitative measure that can be integrated into clinical decision-making and risk stratification. Emerging evidence suggests that personalized approaches, specifically adjusting thresholds and response criteria based on individual frailty assessments, may substantially enhance the performance of EWS, potentially leading to more effective interventions, improved clinical outcomes and optimized utilization of healthcare resources [22,23].

This study was conceptualized as a descriptive, exploratory analysis. We aimed at describing the relationship between NEWS2 and frailty scores in elderly IHCA patients, with the hypothesis that low NEWS2 scores could be associated with high frailty burden that could precipitate in IHCA.

## 2. Materials and Methods

### 2.1. Study Design and Setting

This monocentric, observational, retrospective study was performed in the Anesthesia and Intensive Care Unit of the University Hospital of Siena, Italy, between January 2022 and January 2024. This study was conceptualized as a descriptive, exploratory analysis about the association between NEWS2 and frailty indices, in accordance with the Strengthening the Reporting of Observational Studies in Epidemiology (STROBE) guidelines [24].

### 2.2. Ethical Approval

This study was approved by the Local Ethical Committee (CE Area Toscana Sudest, Protocol ID 29280).

### 2.3. Study Population and Data Collection

All adult patients (≥18 years) admitted to general medical or surgical wards who experienced an in-hospital cardiac arrest (IHCA) with subsequent activation of the Rapid Response System (RRS)/Medical Emergency Team (MET) during the study period were eligible for inclusion. Patients younger than 18 years at the time of the event were excluded.

Data were retrospectively collected from electronic medical records and RRS/MET activation forms. We collected data on demographics, pre-existing chronic diseases, CCI, CFS, BI, and the last NEWS2 score available in the medical records preceding the cardiac arrest. Mortality data and length of hospital stay were also recorded.

### 2.4. Score Calculation

This study evaluated the last available NEWS2 score recorded in the electronic medical records of all adult patients (≥18 years old) admitted to general medical or surgical wards who experienced an IHCA with subsequent activation of the RRS/MET between January 2022 and January 2024.

NEWS2 is a clinical assessment tool based on six physiological variables: respiratory rate, oxygen saturation, systolic blood pressure, heart rate, body temperature and level of consciousness (including new-onset confusion). Each variable is assigned a score ranging from 0 to 3, with an additional 2 points allocated to patients receiving supplemental oxygen. The tool was designed to improve the early recognition of clinical deterioration in acutely ill patients, with scores of 5–6 or higher serving as a threshold for urgent clinical intervention. According to the NEWS2 classification, patients were initially stratified into four categories (Stable, Potentially Unstable, Unstable and Critical) [11,25]. Depending on severity, interventions may include the activation of the attending ward physician or the RRS/MET (Table 1) [26].

Considering that the Potentially Unstable and Unstable categories were not discriminatory for emergency team activation (RRS/MET) in our patient population, and given that both levels require urgent medical evaluation, these two categories were merged into a single group. Thus, we obtained three categories: Stable patients (NEWS2-A), Potentially Unstable + Unstable patients (NEWS2-B), and Critical patients (NEWS2-C). This aggregation reduced subgroup fragmentation while preserving the clinical interpretability of the analysis.

In parallel with NEWS2, the CFS was employed to characterize patients’ baseline functional reserve and frailty status. This nine-point scale evaluates functional status based on independence in activities of daily living and overall health condition during the two weeks preceding the onset of acute illness. Scores of 1–3 correspond to individuals who are classified as very fit, fit or managing well, whereas a score of 4 reflects very mild frailty. Values ranging from 5 to 8 indicate increasing severity of frailty (mild, moderate, severe and very severe), typically associated with a need for assistance in everyday activities. A score of 9 is reserved for terminally ill patients. The scale is straightforward to administer, requires minimal time and is therefore suitable for use in acute care settings [27,28,29].

Complementary to frailty assessment, functional independence was evaluated using the BI. This ordinal scale measures the ability to perform ten basic activities of daily living, including feeding, bathing, grooming, dressing, bowel and bladder control, toilet use, transfers, mobility and stair climbing. Scores range from 0 to 100, with lower values indicating greater functional impairment and dependence, and higher values reflecting greater independence. The BI is widely validated, easy to apply in both clinical and research settings, and provides valuable insights into patients’ baseline autonomy and recovery potential. The BI was administered by nursing staff at the time of ward admission, drawing on both medical history and the patient’s observable clinical status on admission to derive the final score.

Additionally, to better account for the impact of chronic health conditions on outcomes, a comorbidity burden assessment was performed using a validated comorbidity index (Charlson Comorbidity Index, CCI). The CCI assigns weighted scores from 1 to 6 to a range of chronic diseases (e.g., myocardial infarction—1 point, diabetes with end-organ damage—2 points, chronic kidney disease—2 points, malignancies—6 points, etc.), producing a cumulative score. Higher CCI scores indicate greater comorbidity burden and are associated with increased mortality (Table 2) [15,30]. The CCI was calculated for each patient using diagnostic information available in the electronic medical records prior to the IHCA event, allowing for its inclusion in subsequent analyses as an independent variable. Patients younger than 18 years at the time of the event were excluded.

### 2.5. Study Outcomes

The primary endpoint of this descriptive study was to evaluate whether combining the NEWS2 score with frailty indices (BI, CCI and CFS) could enhance the characterization of frail patients at risk of IHCA.

### 2.6. Statistical Analysis

Binary variables were expressed as counts (percentage) and continuous variables as means ± standard deviation or medians with interquartile range (25th to 75th percentiles), depending on the normality of distribution. The Shapiro–Wilk test, histograms, and normal quantile plots were used to verify the normality of distribution of continuous variables. Associations between continuous variables and NEWS2 severity categories were assessed using the non-parametric Kruskal–Wallis test. When significant, post hoc pairwise comparisons were performed with the Dwass–Steel–Critchlow–Fligner method. For categorical variables (e.g., age groups, comorbidities), contingency tables and Chi-square or Fisher’s exact tests were used as appropriate. Data were analyzed using software Jamovi (version 2.6.26) software. A *p*-value < 0.05 was considered statistically significant.

## 3. Results

**Figure 1:**
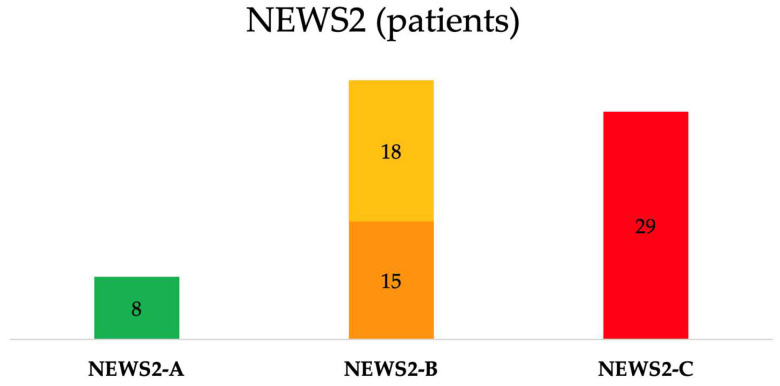
Patients’ distribution based on NEWS2 classification. NEWS2-A identifies Stable patients (*N* = 8), NEWS2-B combines Potentially Unstable (*N* = 18) and Unstable (*N* = 15) patients, and NEWS2-C includes Critical patients (*N* = 29) requiring immediate high-level response.

A total of 75 patients who experienced IHCA with RRS/MET activation were identified. Three of them lacked data on BI, CCI and CFS, and two of them had missing outcome information. As such, the final cohort included 70 IHCA subjects (mean age of 76.9 ± 11.0 years, and 56% were male). Given the small sample size and single-center design, our findings should be interpreted as descriptive and exploratory. The majority (N = 50, 71%) of patients were admitted to medical wards; 20 (29%) of them were in surgical wards. Based on the last NEWS2 recorded before the IHCA, the mean NEWS2 score before IHCA in the overall population was 6.0 ± 3.5, distributed as follows: 1.8 ± 1.3 in the Stable group (*N* = 8, 11%), 3.2 ± 1.2 in the Potentially Unstable group (*N* = 18, 26%), 5.1 ± 1.3 in the Unstable group (*N* = 15, 22%) and 9.4 ± 2.4 in the Critical group (*N* = 29, 41%) (Table 3, Figure 1).

Following the reclassification applied in this study, 41% of patients (N = 29) were included in the NEWS2-C group, 48% (N = 33) in the NEWS2-B group, and 11% (N = 8) in the NEWS2-A group (Table 4, Figure 1).

BI values showed marked variation across NEWS2 categories: NEWS2-A patients had substantially higher scores (64.4 ± 35.5) compared with NEWS2-B (24.7 ± 32.7) and NEWS2-C (11.5 ± 26.4; *p* < 0.01) patients; in particular, the NEWS2-A subgroup had significantly higher BI values than the other two. By contrast, CFS significantly increased among the NEWS2 groups, with an increase from 4.3 ± 1.8 in the NEWS2-A group to 6.14 ± 1.75 in the NEWS2-C group (*p* < 0.01), with results similar to the BI for subgroup comparisons. CCI was 6.5 ± 2.7 in the overall population, non-significantly ranging from 5.4 ± 3.1 in the NEWS2-A group to 7.1 ± 3.2 in the NEWS2-C group (*p* = 0.43). Age was not significantly different among the subgroups (Table 2).

**Figure 2:**
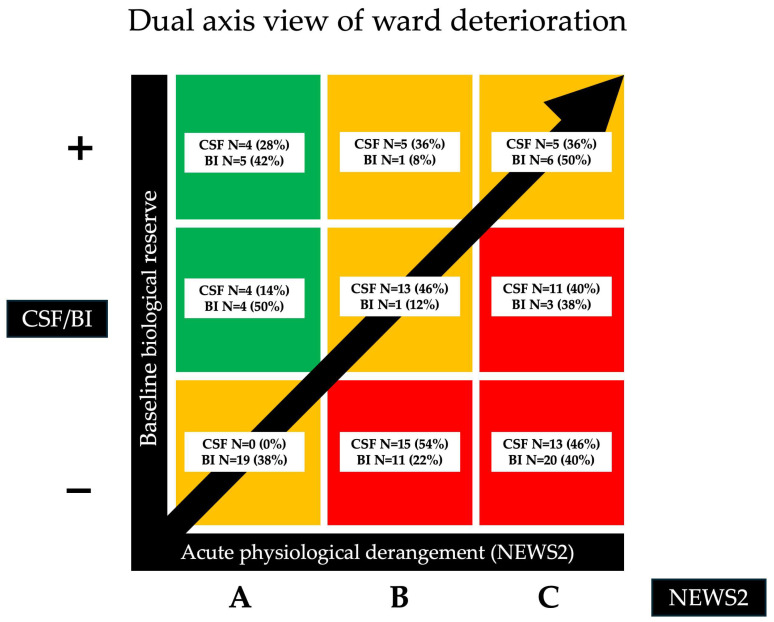
Dual-axis diagram showing the distribution of patients according to aggregated NEWS2 severity (horizontal axis: increasing derangement) and baseline biological reserve (vertical axis: higher to lower reserve). Low reserve—Clinical Frailty Scale (CFS) 7–10 and Barthel Index (BI) < 30; intermediate reserve—CFS 5–6 and Barthel Index (BI) 30–59; high reserve—CFS 1–4 and Barthel Index (BI) ≥ 60. Each cell indicates the number and percentage of patients (N, %) with specific combinations of NEWS2 and reserve: High CFS and BI reserve: CFS N = 4, 28% and BI N = 5, 42% (NEWS2-A); CFS N = 5, 36% and BI N = 1, 8% (NEWS2-B); CFS N = 5, 36% and BI N = 6, 50% (NEWS2-C). Intermediate CFS and BI reserve: CFS N = 4, 14% and BI N = 4, 50% (NEWS2-A); CFS N = 14, 46% and BI N = 1, 12% (NEWS2-B); CFS N = 11, 40% and BI N = 3, 38% (NEWS2-C). Low CFS and BI reserve: CFS N = 0, 0% and BI N = 19, 38% (NEWS2-A); CFS N = 15, 54% and BI N = 11, 22% (NEWS2-B); CFS N = 13, 46% and BI N = 20, 40% (NEWS2-C).

The distribution of the NEWS2 subgroups (i.e., acute physiological derangement) in relation to CFS and BI (i.e., baseline reserve) is represented using a dual-axis diagram in Figure 2.

## 4. Discussion

In this descriptive, exploratory study, we examined a cohort of 70 hospitalized patients who experienced IHCA, with the aim of assessing whether the combination of the NEWS2 score with frailty indices such as the BI, CCI and CFS could enhance the characterization of the pre-arrest condition of elderly IHCA patients. To date, few studies have reported specifically on the performance of NEWS2 scores in frail elderly patients for identifying subjects who would potentially face IHCA [28,30,31,32]. Patients in our cohort were heterogeneously distributed across NEWS2 strata prior to arrest (Stable, Potentially Unstable, Unstable and Critical), suggesting that reliance on physiological scores alone may delay RRS activation and hinder early interception of the patients who experience IHCA. Although the most fragile patients tended to cluster in the Critical group, others who experienced IHCA were classified as Stable or Potentially Unstable/Unstable, suggesting that NEWS2 alone underestimates the risk. Because the Potentially Unstable and Unstable tiers were heterogeneous and operationally non-discriminatory for emergency activation in our setting, we merged them into a single group (NEWS2-B), yielding three bands for analysis: Stable (NEWS2-A), Potentially Unstable + Unstable (NEWS2-B) and Critical (NEWS2-C) [33]. This approach reduced subgroup fragmentation and emphasized the need to integrate frailty and functional indices to capture vulnerability, not reflected by vital signs alone. Our data showed differences in functional status and frailty across NEWS2-A/B/C, whereas age and CCI provided limited additional information. This pattern aligns with an expanding body of research in which frailty and function outperform comorbidity combined with evidence indicating that NEWS2 improves the characterization of such patients versus NEWS2 alone [34,35]. However, the limited sample size (70 patients, all with IHCA) may have reduced the ability to detect a true discriminative effect of CCI.

Our findings also suggested that CFS and BI were significantly different across NEWS2 categories, reflecting susceptibility to acute clinical conditions in the 24 h prior to arrest. In practice, integrating CFS and BI with NEWS2 may facilitate earlier interception of at-risk patients and prompt timely RRS evaluation before IHCA occurs. In particular, patients with a high frailty burden but only modest physiological derangements may have reduced tolerance to acute stressors, predisposing them to clinical deterioration and cardiac arrest. Compared with other older patients experiencing similar acute abnormalities but lower frailty, these individuals may be at higher risk despite relatively preserved vital signs. This vulnerability should alert RRS/MET to the need for heightened surveillance, as reliance on standard vital parameters alone may fail to identify their increased risk of cardiac arrest. However, as our study included only patients who experienced IHCA and lacked a comparator group, it does not allow for the assessment of true risk, prediction, or discrimination. Thus, our findings generated only hypotheses about frailty/NEWS2 relationships rather than establishing predictive or preventive utility. Moreover, our study cannot establish a specific cutoff value for any single score, nor can it validate any score combination to predict IHCA. Instead, these measures may act as early alerts prompting RRS assessment, enabling experienced clinicians to recognize patients whose condition is worsening. A prospective model developed by Lo Conte et al. on a large cohort of patients, using logistic regression analysis, showed that integration of the BI with NEWS2 improved the detection of high-risk patients and outperformed NEWS2 alone in predicting both deterioration and in-hospital mortality [36]. In contrast, Chung et al., in a multicenter retrospective study, reported that adding the CFS to NEWS2 significantly improved 30-day mortality prediction in older emergency department patients. In addition, Wretborn et al., in a secondary analysis of a prospective observational study, demonstrated that combining CFS with vital sign-based early warning systems yielded superior risk stratification for short- and long-term mortality compared to physiology alone [34,37]. However, in their study, the CCI showed no significant association with NEWS2, suggesting that chronic comorbidities may not sufficiently capture short-term vulnerability in acutely deteriorating patients. Finally, in a retrospective study including 478 COVID-19 patients, it was found that although both NEWS2 and CCI were associated with mortality, the incremental value of CCI was limited, particularly in older populations with a uniformly high comorbidity burden [38]. From a clinical perspective, these findings support a dual-axis model of ward deterioration: acute physiological derangement (NEWS2) and baseline biological reserve (as captured by frailty and functional status). Embedding frailty measures such as CFS and BI into escalation algorithms, particularly when NEWS2 is intermediate (NEWS2-B), could sharpen triage, reduce missed deterioration in the frail, and prevent unnecessary RRS activations. Such integration might allow for earlier activation of the RRS/MET even at lower NEWS2 thresholds (e.g., NEWS2-A), especially in patients whose clinical fragility is not fully captured by vital signs alone.

Our study has some strengths: it addresses an understudied but clinically relevant population (elderly hospitalized patients with IHCA), and integrates multiple dimensions of vulnerability (NEWS2, CFS, BI, and CCI), demonstrating their differential association with acute deterioration. However, these strengths should be tempered by several limitations. The retrospective, single-center design and relatively small sample constrain precision and generalizability. The absence of a control group, as only patients who experienced IHCA were included, prevents comparison with patients who did not experience arrest and thus precludes any estimation of real risk or incremental predictive value. In addition, the use of the last recorded NEWS2 introduces a timing bias, which may fail to capture the true peri-arrest physiological trajectory. Finally, because of the observational design, causal inferences cannot be drawn, and our findings should be interpreted as descriptive and hypothesis-generating only. Prospective, multicenter studies, with larger sample sizes, ideally incorporating trend-based early warning metrics and frailty-augmented escalation algorithms, are warranted to validate these descriptive findings and to inform tailored intervention strategies. Future research should aim to develop and validate frailty-informed early warning models within RRSs, integrating physiological, functional and frailty data to improve early recognition and tailored escalation of care in older, vulnerable patients. Until such evidence becomes available, the clinical implementation of frailty-informed escalation thresholds should be approached with caution.

## 5. Conclusions

In this descriptive and exploratory study, NEWS2 values showed substantial variability among older patients with in-hospital cardiac arrest. Integrating NEWS2 with frailty assessment represents a hypothesis-generating approach to identify older adults with reduced physiological reserve and limited tolerance to acute deterioration, including in-hospital cardiac arrest. Larger prospective, multicenter studies are warranted to validate this approach’s clinical utility.

## Figures and Tables

**Table 1:** NEWS2 score description.

Category	NEWS2	Medical Response
Stable	0	Standard monitoring
Potentially Unstable	1–4	Increase monitoring frequency
Unstable	5–6 or single score = 3	Urgent medical evaluation
Critical	≥7	Activate RRS/MET

NEWS2 scoring chart based on respiratory rate (RR), oxygen saturation (SpO_2_), supplemental oxygen use, systolic blood pressure (SBP), heart rate (HR), consciousness level (ACVPU) and temperature. Total score indicates clinical risk and guides escalation of care [24].

**Table 2:** Baseline characteristics of patients according to NEWS2-A/B/C.

Variable	NEWS2-A (*N* = 8)	NEWS2-B (*N* = 33)	NEWS2-C (*N* = 29)	*p*-Value
CCI	5.4 ± 3.1	6.2 ± 2.0	7.1 ± 3.2	0.43
BI	64.4 ± 35.5	24.7 ± 32.7	11.6 ± 26.4	0.01
CFS	4.3 ± 1.8	6.1 ± 1.4	6.1 ± 1.8	0.01
Age (years)	70.6 ± 12.4	76.7 ± 9.2	79.0 ± 12.2	0.10

Baseline characteristics of patients according to NEWS2-A/B/C. Kruskal–Wallis test: post hoc (Dwass–Steel–Critchlow–Fligner): BI (higher BI scores reflect greater independence and less frailty) NEWS2-A group > NEWS2-B group (*p* < 0.01), NEWS2-A group > NEWS2-C group (*p* < 0.01); no difference between B and C. CFS (higher CFS scores reflect greater frailty and less independence): NEWS2-A group < Group B (*p* = 0.02), NEWS2-A group < NEWS2-C (*p* = 0.02); no difference between B and C. CCI (higher CCI scores are associated with increased mortality) and Age: no significant differences among groups.

**Table 3:** Characteristics of the study population.

Variable	Overall (N = 70)	NEWS2 Stable (N = 8)	NEWS2 Potentially Unstable (N = 18)	NEWS2 Unstable (N = 15)	NEWS2 Critical (N = 29)
Age (years)	76.9 ± 11.0	70.6 ± 12.4	76.4 ± 9.0	77.0 ± 9.8	79.00 ± 12.2
Sex male N (%)	39 (55.7%)	5 (62.5%)	8 (44.4%)	7 (46%)	19 (65.5%)
Length of hospital stay (days)	13.8 ± 15.8	18.5 ± 27.0	12.6 ± 12.6	17.0 ± 16.2	11.6 ± 13.7
Arterial hypertension N (%)	38 (54.3%)	2 (25.0%)	10 (55.6%)	9 (60.0%)	17 (58.6%)
Diabetes N (%)	23 (32.9%)	0 (0%)	7 (38.9%)	5 (33.3%)	13 (44.8%)
Heart failure N (%)	14 (20.0%)	0 (0%)	4 (22.2%)	4 (26.7%)	8 (27.6%)
COPD/Asthma N (%)	12 (17.1%)	1 (12.5%)	5 (27.8%)	1 (6%)	5 (17.2%)
Stroke/TIA N (%)	13 (18.6%)	1 (12.5%)	2 (11.1%)	3 (20%)	7 (24.1%)
Liver disease N (%)	9 (12.9%)	0 (0%)	3 (16.7%)	2 (13%)	4 (13.8%)
Neoplasia N (%)	21 (30.0%)	2 (25.0%)	5 (27.8%)	3 (20%)	11 (37.9%)
Renal failure N (%)	17 (24.3%)	2 (25.0%)	5 (27.8%)	2 (13%)	8 (27.6%)
CCI	6.5 ± 2.7	5.4 ± 3.1	6.5 ± 1.8	5.9 ± 2.3	7.1 ± 3.2
BI	23.8 ± 34.1	64.4 ± 35.5	31.4 ± 34.0	16.6 ± 30.1	11.6 ± 26.4
CFS	5.9 ± 1.7	4.3 ± 1.8	5.9 ± 1.3	6.4 ± 1.5	6.1 ± 1.8
Last NEWS2	6.0 ± 3.5	1.8 ± 1.3	3.2 ± 1.2	5.1 ± 1.3	9.4 ± 2.4
Defibrillable rhythm N (%)	5 (7.1%)	3 (37.5%)	1 (5.6%)	0 (0%)	1 (3.4%)
ROSC N (%)	16 (22.9%)	3 (37.5%)	4 (22.2%)	3 (20%)	6 (20.7%)
Death N (%)	65 (92%)	5 (62%)	16 (88%)	15 (100%)	29 (100%)

Characteristics of the study population divided into Stable patients, Potentially Unstable patients, Unstable patients and Critical patients.

**Table 4:** Characteristics of the study population divided into three groups.

Variable	NEWS2-A (*N* = 8)	NEWS2-B (*N* = 33)	NEWS2-C (*N* = 29)
Age (years)	70.6 ± 12.4	76.7 ± 9.2	79.0 ± 12.2
Sex male N (%)	5 (62.5%)	15 (45.5%)	19 (65.5%)
Length of hospital stay (days)	18.5 ± 27.0	14.6 ± 14.3	11.6 ± 13.7
Arterial hypertension N (%)	2 (25.0%)	19 (57.6%)	17 (58.6%)
Diabetes N (%)	0 (0.0%)	10 (30.3%)	13 (44.8%)
Heart failure N (%)	0 (0.0%)	6 (18.2%)	8 (27.6%)
COPD/Asthma N (%)	1 (12.5%)	6 (18.2%)	5 (17.2%)
Stroke/TIA N (%)	1 (12.5%)	5 (15.2%)	7 (24.1%)
Liver disease N (%)	0 (0.0%)	5 (15.2%)	4 (13.8%)
Neoplasia N (%)	2 (25.0%)	8 (24.2%)	11 (37.9%)
Renal failure N (%)	2 (25.0%)	7 (21.2%)	8 (27.6%)
Last NEWS2	1.8 ± 1.3	4.1 ± 1.6	9.5 ± 2.4
Defibrillable rhythm N (%)	3 (37.5%)	1 (3.0%)	1 (3.4%)
ROSC N (%)	3 (37.5%)	7 (21.2%)	6 (20.7%)
Death N (%)	5 (62%)	31 (93%)	29 (100%)

Characteristics of the study population divided into three groups: NEWS2-A includes Stable patients, NEWS2-B includes Potentially Unstable + Unstable patients, and NEWS2-C includes Critical patients.

## Data Availability

The data that support the findings of this study are available from the corresponding author upon reasonable request.
